# Extraocular Sebaceous Carcinoma of the Scalp in an Elderly Patient

**DOI:** 10.7759/cureus.107209

**Published:** 2026-04-17

**Authors:** Aliki Vaia Rompou, Andreas Nikolaos Dafnis, Evangelia Boti, Georgios Kitsakis, Dimitris P Korkolis

**Affiliations:** 1 Surgical Oncology, General Anticancer and Oncology Hospital of Athens "Saint Savvas", Athens, GRC; 2 Pathology and Laboratory Medicine, General Anticancer and Oncology Hospital of Athens "Saint Savvas", Athens, GRC

**Keywords:** cutaneous malignancy, elderly patient, extraocular sebaceous carcinoma, scalp tumor, sebaceous carcinoma, wide local excision

## Abstract

Sebaceous carcinoma (SC) is a rare and aggressive malignant neoplasm of the sebaceous glands, most commonly arising in the periocular region. Extraocular SC, particularly when located on the scalp, is a rare condition that is often misdiagnosed, resulting in delays in treatment.

An 85-year-old woman presented to the outpatient clinic with a slowly enlarging scalp mass. Initial surgical excision was performed, and histopathological examination confirmed SC. The patient subsequently underwent wide local excision and comprehensive staging imaging, which revealed no residual disease or metastatic spread. She was therefore managed with close clinical follow-up.

Extraocular SC is an aggressive neoplasm with a significant risk of local recurrence and metastasis, particularly when arising on the scalp, where it may be misdiagnosed as other cutaneous malignancies. Accurate diagnosis relies on histopathological evaluation supported by immunohistochemical analysis. Complete surgical excision with clear margins, including wide local excision or Mohs micrographic surgery, remains the cornerstone of treatment. Early recognition and timely intervention are critical determinants of prognosis.

SC of the scalp is a rare but potentially life-threatening malignancy. Inclusion of this entity in the differential diagnosis of atypical scalp lesions in elderly patients, along with prompt biopsy and appropriate surgical management, is essential to optimize clinical outcomes.

## Introduction

Sebaceous carcinoma (SC), first described in 1956 by Straatsma, is a rare malignant neoplasm arising from the sebaceous glands [1]. Although it can occur anywhere on the body, it most commonly affects the periocular region, particularly the eyelids [2,3]. SC represents approximately one percent of eyelid tumors and about 4.7 percent of malignant epithelial eyelid tumors [4].

Extraocular SC is uncommon, with the scalp representing a particularly rare site of involvement [2,5]. These tumors frequently mimic benign or inflammatory lesions, which may contribute to delayed diagnosis and inappropriate initial management [3,6]. Given its potential for local recurrence and metastatic spread, early recognition and accurate histopathological diagnosis are essential.

## Case presentation

**Figure 1:**
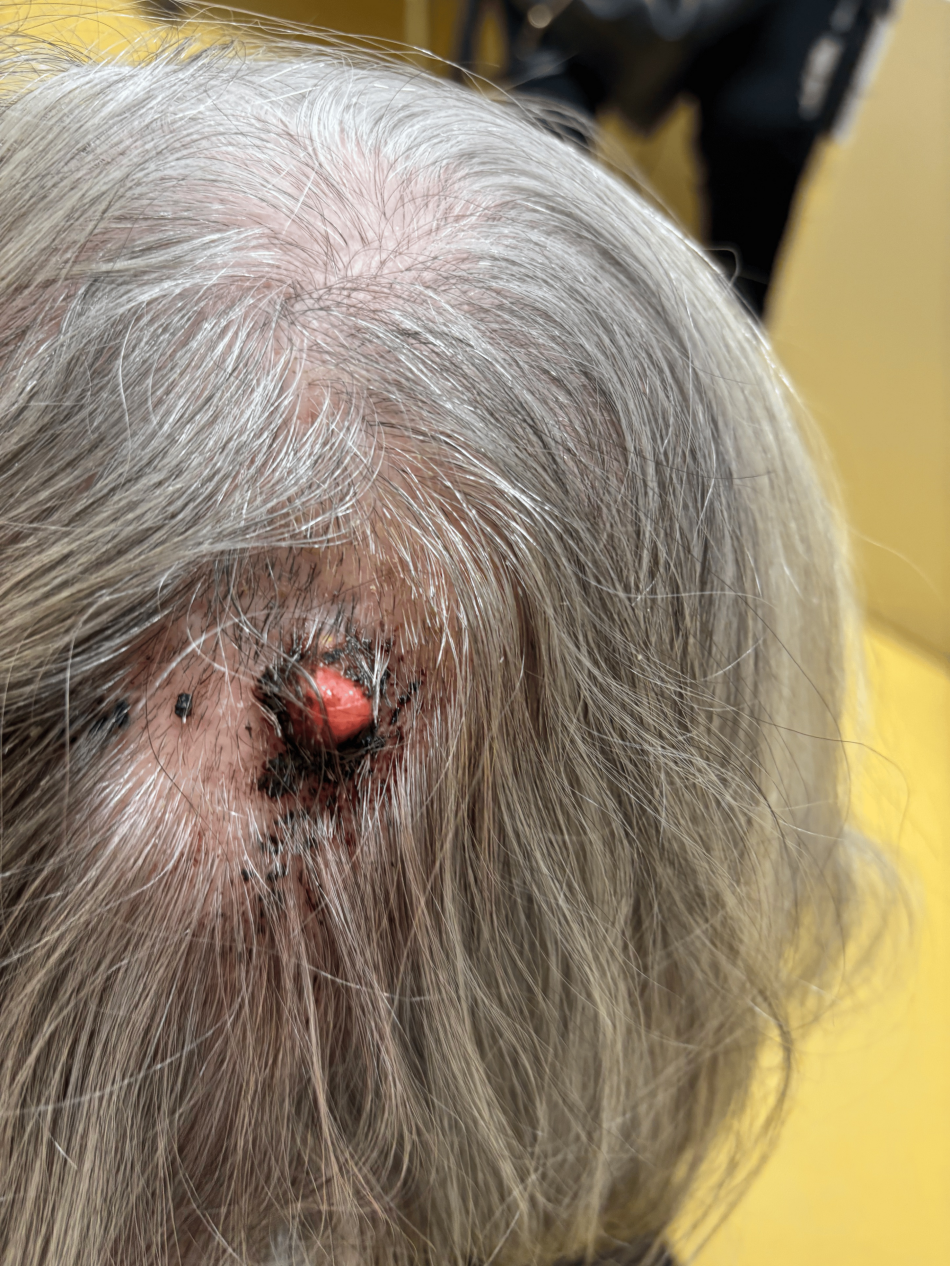
Clinical appearance of the scalp lesion Photograph showing a  friable lesion in  the scalp prior to surgical excision. The lesion measured approximately 1.2 × 1.2 cm and demonstrated irregular borders with surface crusting.

An 85-year-old woman presented to the outpatient clinic with a six-month history of a progressively enlarging lesion in the parieto-occipital region of the scalp (Figure 1). The patient reported intermittent bleeding and crusting related to repeated local trauma but denied pain, weight loss, or other systemic symptoms. Her medical history was notable for arterial hypertension, dyslipidemia, and type 2 diabetes mellitus. There was no known personal or family history suggestive of an inherited cancer syndrome and no known exposure to chemicals.

Clinical examination revealed an erythematous, nodular, and friable lesion with focal crusting measuring approximately 1.2 × 1.2 cm with ill-defined margins. No palpable cervical or occipital lymphadenopathy was identified.

**Figure 2:**
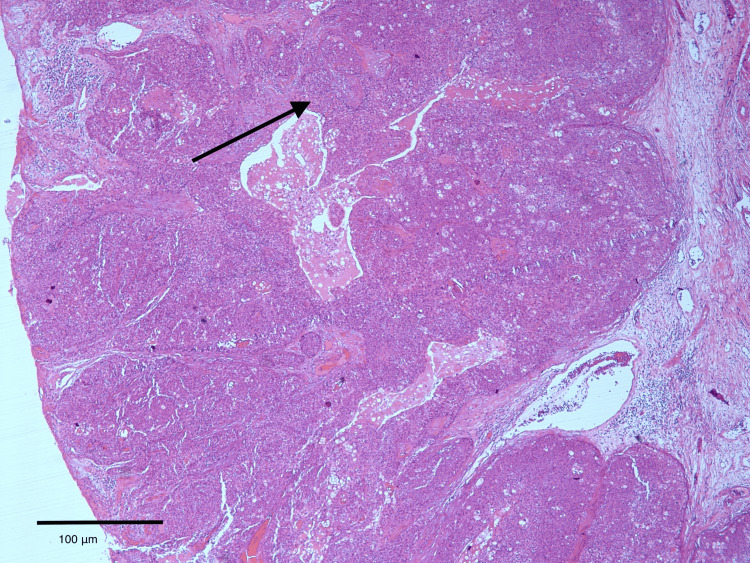
Histopathological examination demonstrating dermal lobules of sebaceous carcinoma composed of atypical tumor cells. The arrow indicates a representative tumor lobule. Scale bar: 100 μm Organoid proliferations comprising dermal lobules of variably atypical polygonal cells with fibrovascular stroma that typically lacks desmoplasia. Histologic findings consistent with sebaceous carcinoma (H&E stain, x10).

**Figure 3:**
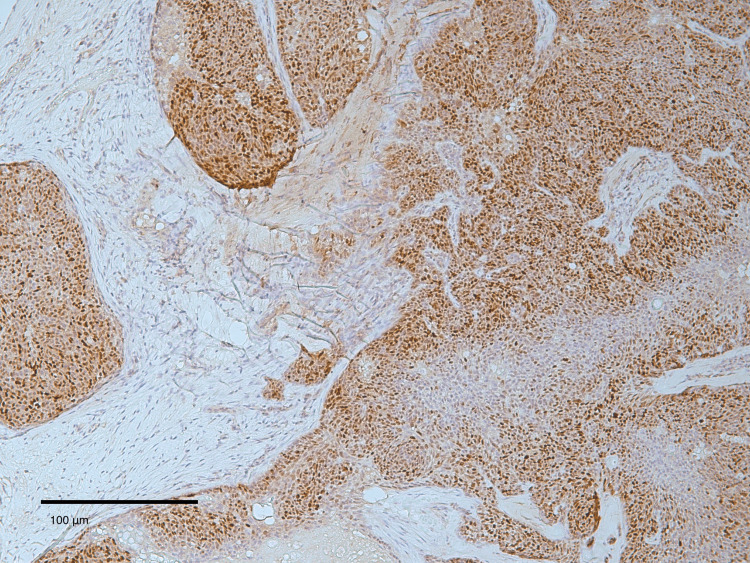
Immunohistochemical features of the tumor Immunohistochemical staining for androgen receptor (positive) showing strong nuclear positivity in the neoplastic cells, supporting the diagnosis of sebaceous carcinoma.

The lesion was initially surgically excised with histologically clear but close margins, with the closest peripheral margin measuring 1 mm, the opposite peripheral margin 2 mm, and the deep margin 2 mm. Histopathological evaluation demonstrated features consistent with SC with epidermal ulceration (Figure 2). Immunohistochemical analysis showed positivity for androgen receptor, BCL-2, EMA (focal), and p63, with negativity for MOC31 and CD34, supporting sebaceous differentiation and confirming the diagnosis (Figure 3). Immunohistochemical screening for mismatch repair (MMR) proteins (MLH1, MSH2, MSH6, PMS2) was not performed.

In view of the close margins, the patient subsequently underwent wide local excision with predefined circumferential margins of 1 cm, extending to the galea as the deep margin, to ensure complete tumor clearance.

Computed tomography of the head and neck was performed for staging purposes and demonstrated no evidence of bone invasion or regional lymph node involvement. The patient was managed with close clinical follow-up, and no signs of local recurrence or metastatic disease were observed at the six-month review.

## Discussion

Extraocular SC is a rare but potentially aggressive cutaneous malignancy with a documented risk of local recurrence and regional or distant metastasis [3,6]. The scalp represents an uncommon site of presentation, and lesions in this location may clinically resemble basal cell carcinoma, squamous cell carcinoma, or benign adnexal tumors, leading to diagnostic delay [2,6]. Early recognition and histopathological confirmation are therefore essential to avoid undertreatment.

Reported risk factors include advanced age, prior radiation exposure, immunosuppression, and inherited cancer syndromes [3]. Muir-Torre syndrome is characterized by sebaceous neoplasms occurring in association with internal malignancies and is considered a phenotypic variant of Lynch syndrome [3]. In the present case, although there were no clinical features suggestive of a hereditary syndrome, advanced age and cumulative sun exposure were considered potential contributing factors. Although no personal or family history suggestive of an inherited cancer syndrome was identified, current oncologic practice supports routine immunohistochemical screening for mismatch repair (MMR) protein deficiency in extraocular sebaceous neoplasms. In the present case, MMR immunohistochemistry was not performed, representing a limitation of the diagnostic workup and an important consideration for future cases.

Histopathological examination remains the cornerstone of diagnosis. Immunohistochemical markers such as adipophilin, epithelial membrane antigen, androgen receptor, and p63 may assist in confirming sebaceous differentiation, particularly in poorly differentiated tumors [7]. Accurate interpretation is critical, as extraocular lesions may demonstrate variable morphologic patterns.

Complete surgical excision with histologically clear margins is the primary treatment for localized disease. Both wide local excision and Mohs micrographic surgery are accepted approaches, depending on tumor size and location [3,8]. In the present case, initial excision with close margins (1-2 mm) necessitated re-excision with wider oncologic margins (1 cm circumferentially, extending to the galea), which is consistent with standard surgical practice to minimize the risk of local recurrence. Adjuvant radiotherapy may be considered in selected cases involving lymph node metastasis, incomplete resection, or recurrent disease [9,10]. Given the potential for late recurrence or metastasis, long-term clinical surveillance is recommended [3]. A structured follow-up strategy is advisable, including clinical examination of the surgical site and regional lymph nodes every 3-6 months during the first two years, followed by annual assessments thereafter. In addition, periodic imaging, consisting of ultrasound of regional lymph node basins or cross-sectional imaging when clinically indicated, may be considered to facilitate early detection of recurrence or metastatic disease.

## Conclusions

SC of the scalp represents a rare and potentially aggressive cutaneous malignancy that may present with nonspecific clinical features, leading to delayed diagnosis. This case highlights the importance of maintaining a high index of suspicion for SC when evaluating atypical or nonhealing scalp lesions in elderly patients. Accurate histopathological assessment supported by immunohistochemical analysis is essential for establishing the diagnosis and guiding management. Complete surgical excision with clear margins remains the cornerstone of treatment and is associated with favorable short-term outcomes when performed promptly. Given the risk of local recurrence and metastatic spread, long-term clinical surveillance is warranted even in cases without initial evidence of regional or distant disease.
